# How social media shapes urban image and conative tourism behaviour among Chinese Generation Z: an extended SOR model

**DOI:** 10.3389/fpsyg.2026.1713853

**Published:** 2026-06-17

**Authors:** Xin Huang, Jinping Lin, Yiyi Xu, Peng Li, Siyu Zhuo, Ziyao Chen

**Affiliations:** 1 College of Artificial Intelligence, Putian University, Putian, China; 2 School of Culture and Communication, Putian University, Putian, China; 3 School of Animation and Digital Arts, Communication University of China, Beijing, China

**Keywords:** empathy, Generation Z, social media, SOR framework, urban conative behaviour, urban image

## Abstract

This research investigates the influence of social media material on urban image and urban conative behaviour across Chinese Generation Z. Utilising the Stimulus–Organism–reaction (SOR) framework, it formulates an enhanced model wherein real-time content perception (RTCP) and empathy function as dual organism-level mechanisms connecting external content inputs to urban image and subsequent behavioural response. Three types of social media stimuli are looked at: electronic word-of-mouth (eWOM), content quality (CQ), and location-based user-generated content (LB-UGC). Data were gathered from 550 Chinese Generation Z social media users, and structural equation modelling was utilised to evaluate the potential associations. The findings indicate that both RTCP and empathy substantially mediate the influence of social media stimuli on urban image and urban conative behaviour. The effects differ among stimulus types: CQ and LB-UGC exhibit a stronger correlation with RTCP, while eWOM demonstrates a closer association with empathy. Urban image also serves as a significant perceptual process that converts interior psychological feelings into urban conative behaviour. The research enhances the literature by augmenting the SOR framework with a unified process that integrates cognitive assessment and affective response in the construction of urban images on social media. It also has real-world uses for city branding and digital communication by showing how important timely, location-based, and emotionally engaging content is for changing younger users’ impressions and reactions to destinations.

## 1 Introduction

Tourism growth has made urban images easier to create, circulate, and reinforce (Haponenko et al., 2024). Research on urban image and tourism behaviour has also expanded rapidly, much of it in connection with destination branding (Rather, 2021; Liang and Arana Del Carpio, 2025). Brand communication channels play an important role in shaping travel-related responses (Tong et al., 2022), and scholars have examined how urban image relates to motives associated with destination characteristics (Li et al., 2020). At the same time, the processes through which urban images are formed and shared are changing. New media technologies, especially their constant updating and routine use in everyday life, are reshaping how urban images are produced and consumed (Khalid et al., 2024). Even so, the literature still says relatively little about how users perceive destination cities through social media, particularly through short-video platforms and location-based user-generated content (LB-UGC) (Zhang et al., 2023; Li et al., 2021a,b).

Current evidence suggests that urban image is no longer shaped mainly by official promotional material. Instead, it is increasingly produced through interactive digital content environments (Yin et al., 2023; Tellis et al., 2019). User-generated content (UGC) is crucial to this process, since consumers frequently see it as more genuine, immediate, and participatory compared to institutional communication (Khan M N. et al. (2021); Enrique Bigné et al., 2009; Lu et al., 2020). Location-based social media platforms such as Douyin/TikTok and Instagram further intensify this process by attaching geotags and other contextual cues to content (Li et al., 2023; Dang et al., 2024; Jiang et al., 2022). This can be seen in urban exploration challenges on short-video platforms, immersive scenario design that encourages exploration, and real-time AR maps that support spatial recognition through interactive features (Chen et al., 2020; Sun et al., 2025). Constant streams of updated information, including live coverage of urban events or incidents, may further increase both the visibility and complexity of urban image formation (Chen et al., 2020). Research also shows that electronic word-of-mouth (eWOM) often diffuses more widely than conventional word-of-mouth, whether the message is positive or negative (Cheong et al., 2020; Sutanto and Aprianingsih, 2016). As a result, UGC on digital platforms has become an important source of destination-related information (Molinillo et al., 2020a,b).

Although urban image is widely treated as an important construct in explaining tourism behaviour, loyalty, and behavioural responses (Rather et al., 2022), most studies still examine new media at a broad level. They typically concentrate on the overarching function of digital media in urban brand communication rather than the particular content attributes that influence users’ psychological responses and subsequent behaviours (Campbell and Connelly, 2020; Li, 2025). Consequently, the impacts of micro-level content attributes, including real-time display, location relevance, narrative structure, and interactive design, are little examined.

This study investigates Generation Z in China, encompassing around 400 million individuals (Zipser et al., 2022; Hong and Liang, 2025). This group is highly significant since its members tend to value experience, individuality, improving their lifestyle, and being well-known on social media. Their consumption patterns in China differ significantly from those of previous generations. For instance, they prefer consumption that is more expressive and facilitated by social media (Suprawan et al., 2024). Because of these features, they are becoming more and more essential in both domestic and overseas markets (Statista, 2023; Zhang et al., 2023). The Chinese tourism market is so big that they are even more essential. Chinese tourists that travel abroad are still one of the greatest groups of visitors in the world (Kumar and Tandon, 2025). Tourism within China is also a key aspect of the economy.

Xiaohongshu and Weibo are particularly essential sources of travel information for Chinese Generation Z and important places for shaping urban images because so many people use new media platforms. Xiaohongshu had more than 120 million monthly active users by 2023, and 70% of them were born in the 1990s or 2000s (QuestMobile, 2023). Weibo said that more than 586 million people used it every month, and that more than 30% of those users were young people (Weibo Corporation, 2023). Because both platforms combine high interactivity, perceived authenticity, and varied content formats, they are likely to shape how young users evaluate tourist destinations and form behavioural intentions (Li et al., 2022; Liu and Liu, 2025; Xu and Zhou, 2025).

Despite extensive work on destination image and tourism behaviour, and growing recognition of the macro-level role of new media in brand communication (Rather et al., 2022; Swain et al., 2024), mportant gaps remain. Existing studies often treat social media content as a uniform stimulus and do not distinguish clearly between the content features that may produce different psychological responses (Smith and Gibson, 2020). For example, although Li et al. (2020) show that user- generated content influences destination image, they do not separate the temporal and spatial dimensions of content in users’ psychological processing. Likewise, Hosany et al. (2020) show that empathy strengthens destination loyalty, but their analysis is grounded in a more traditional media context. It therefore does not explain whether, or how, empathy connects cognitive appraisals ofocation-based, real-time content to later behavioural intentions in an interactive new media environment. Other studies examine variables such as information quality and find that it affects both cognitive and affective destination image (Wei and Chen, 2025). Some focus on user states such as psychological involvement (Zhang et al., 2022). Others propose serial mediation mechanisms involving flow experience and benign envy (Wang et al., 2025), or explain behaviour in terms of social pressure (Liu, 2022). Even so, the literature still lacks a clear account of how contextualised social media content shapes urban image through cognitive and affective processes. By focusing on Chinese Generation Z, this study addresses that gap and offers practical insight into how this group processes social media content and forms urban images.

This study examines various alternative models in the formulation of the theoretical framework, including the stimulus-organism-response (SOR) model, the Technology Acceptance Model (TAM), and the Theory of Planned Behaviour (TPB). TAM and TPB are commonly employed to elucidate technology adoption and rational decision-making (Marikyan et al., 2023); however, they are inadequately equipped for the analysis of the immediate and emotionally charged psychological processes elicited by social media content. TAM emphasises instrumental beliefs, including perceived usefulness and ease of use, whereas TPB concentrates on attitudes, subjective norms, and perceived behavioural control (Ye et al., 2023). Both models are useful, but neither does a good job of capturing the quick, emotional decisions that digital natives make when they interact with immersive and narrative urban content (Dağ et al., 2024). Generation Z in China is a good example because this group is very active on short-video and social media sites like Douyin and Xiaohongshu (Statista, 2023). Their choices about what to buy seem to be based on how they feel, how they interact with others, how real they are, how emotional the stories are, and how quickly they get feedback (Rodrigues et al., 2024; Zhang et al., 2023).

The SOR model offers a more flexible framework for this context because it links external stimuli, internal states, and behavioural responses in a way that accommodates both cognitive appraisal and affective response. Most prioritise cognitive mediation and pay less attention to affective mediation, especially empathy (Bautista et al., 2020). Yet empathy may be a critical mechanism in this setting. Tourism research suggests that empathy strengthens destination loyalty (Hosany et al., 2020; Li and Lai, 2021), but its role within the cognition-affection-behaviour chain in urban image research has not been tested directly.

Against this background, three gaps remain in the literature on urban image formation in social media environments. To begin with, social media content is still frequently treated as a relatively homogeneous stimulus, which obscures the possibility that different content attributes may elicit different psychological responses. A second gap concerns real-time content perception (RTCP). Although real-timeness is often discussed as a feature of digital content, RTCP has rarely been theorised explicitly as an organism-level cognitive appraisal mechanism within the SOR framework, especially in research focusing on Generation Z. A third limitation lies in the way cognitive appraisal and affective response are typically examined. Their joint roles in shaping urban image and subsequent urban conative behaviour remain insufficiently integrated in studies of digitally mediated destination perception.

To address these issues, the present study develops an extended SOR model that differentiates the stimulus layer into content quality (CQ), location-based user-generated content (LB-UGC), and electronic word-of-mouth (eWOM). It further incorporates RTCP and empathy as dual organism-level mediators and treats urban image as a proximal perceptual mechanism linking internal states to behavioural response. On this basis, the study addresses three research questions:

*RQ1*: Which types of social media content stimuli influence Generation Z’s urban image perception through internal psychological mechanisms?
*RQ2*: How does urban image relate to urban conative behaviour among Generation Z?
*RQ3*: What roles do RTCP and empathy play in the transformation of social media stimuli into urban image and behavioural response?

The study contributes to the literature by developing a cognitive-affective dual-pathway model within an extended stimulus-organism-response framework. It addresses a continuing question in tourism research: in new media environments, is tourist behaviour shaped mainly by cognitive evaluation or by affective response? The study argues that these two processes work together rather than separately.

More specifically, content quality, location-based user-generated content, and electronic word-of-mouth are treated as key external stimuli. These stimuli can activate both cognitive appraisal and affective response. Within this framework, real-time content perception and empathy serve as the main psychological mechanisms through which social media content shapes urban image and urban conative behaviour.

This framework helps clarify the underlying process (Gasparino et al., 2024). It shows how specific features of digital content can trigger both reasoned evaluation and emotional engagement among Generation Z users. In doing so, the study offers a more precise explanation of how social media-based urban image is translated into tourism-related behavioural responses (Chen et al., 2022).

## 2 Theory and concepts

### 2.1 Foundations of the SOR model and rationale for its extension

This study utilises the stimulus–organism–response (SOR) model as its primary analytical framework, addressing identified gaps in previous research, and expands it to elucidate the impact of new media-based urban imagery on Generation Z (Li et al., 2024). Research on destination image and media effects has often treated digital content as a single, undifferentiated stimulus, which makes it difficult to explain how specific content features elicit different psychological responses. A further limitation lies in the continuing separation between cognitive and affective explanations of tourism behaviour. Although both perspectives have been developed in parallel, few studies integrate them within a single framework or examine how they work together in digitally mediated settings (Hosany et al., 2020).

At the stimulus level, this study brings together three dimensions of social media-based city content: content quality (CQ), location-based user-generated content (LB-UGC), and eWOM credibility (Chua et al., 2023). CQ is treated as a basic informational feature that shapes cognitive appraisal by influencing how clear, useful, and persuasive content appears to users (Molinillo et al., 2020a,b). LB-UGC adds a spatial dimension. Content that includes geotags, real-time scenes, and context-specific visual material can strengthen users’ sense of place and increase their desire to explore because it makes the destination more concrete and easier to imagine (Zhang et al., 2023; Gao and Li, 2022). eWOM credibility captures the social dimension of the stimulus. In information-saturated social media environments, users rely on credibility cues to filter large volumes of user-generated content and decide what to trust (Leung et al., 2023; Ismagilova, 2023). Taken together, these three constructs represent a multidimensional set of stimuli that combines informational, spatial, and social attributes relevant to Generation Z users.

At the organism level, the study identifies real-time content perception (RTCP) and empathy as the two main mediating mechanisms needed to explain Generation Z’s psychological processing. RTCP is conceptualised as a cognition-based appraisal process through which individuals judge the timeliness, contextual relevance, and practical usefulness of information (De Canio and Endrighi, 2024). When users perceive content as current and contextually relevant, uncertainty may decline and urban conative behaviour may strengthen. Empathy, by contrast, is treated as an affect-based mechanism. It involves emotional projection, identification, and resonance with narrative content, allowing external symbolic material to connect with internal emotional states (Hosany et al., 2020; Yung et al., 2021). Prior research suggests that empathy can be an important predictor of urban conative behaviour and loyalty (Liu et al., 2022). For Generation Z, a cohort strongly oriented towards visual media and emotional engagement (Islam, 2025), these two mechanisms are likely to operate in parallel. RTCP may provide the cognitive grounding that supports emotional engagement, while stronger emotional resonance may in turn increase attention to, and trust in, real-time information (Preston and de Waal, 2002).

Within the Generation Z cohort, high-quality LB-UGC appears especially important in shaping urban image through both RTCP and empathy. The immediacy and spatiotemporal proximity of location-based content can heighten users’ sense of timeliness and increase trust in the content as a representation of current urban life (Espinoza-Figueroa et al., 2025; Qiu et al., 2025). At the same time, affect-rich formats such as narrative vlogs and multisensory short videos can evoke empathy through immersive storytelling and emotional cues, thereby strengthening users’ emotional connection with the destination (Gao et al., 2021; Chi and Phuong, 2022; Tussyadiah and Fesenmaier, 2022). Other content features, including novelty, clarity, and entertainment value, may also increase enjoyment and influence urban conative behaviour indirectly by reducing perceived risk and encouraging affective engagement (Wang and Huang, 2025; Broćić and Miles, 2021; Chen et al., 2014; Tellis et al., 2019). By placing RTCP and empathy at the centre of the framework, the study creates a basis for testing how urban image is formed and how it is translated into urban conative behaviour through the combined effects of cognitive validation and affective resonance (Rodrigues et al., 2024).

At the response level, the study focuses on urban conative behaviour and considers how the distinctive cognitive-affective tendencies of Generation Z may intensify this process. In highly mediated and emotionally charged decision environments, movement from cognitive appraisal to affective attachment is unlikely to be linear. Instead, it appears to be dynamic, and this may be especially pronounced among Generation Z users (Hosany et al., 2020). Having grown up in media environments defined by instant feedback, visual immersion, and continuous interaction, this cohort tends to integrate cognitive and emotional processing closely (Xu and Zhou, 2025). Functional evaluations of a city may quickly generate emotional responses, while strong emotional resonance may also reshape later cognitive judgments (Ladhari and Michaud, 2015). The formation of cognitive and affective images, and the interaction between them, therefore represent key stages in the psychological shift from exposure to urban conative behaviour. Through the mediating role of the organism, new media stimuli contribute to a mental representation of the city that is continuously updated and emotionally charged. The strength and speed of the interaction between cognition and affect within this representation are likely to influence urban conative behaviour directly (Wang et al., 2023). This perspective offers a more precise explanation of how Generation Z moves from basic awareness of a city to a strong intention to visit.

### 2.2 Content quality

Content Quality (CQ) is a fundamental attribute of user-generated content. It includes informational value, credibility, attractiveness, and meeting needs (Molinillo et al., 2020a,b). Research has consistently shown that high-quality content, like in-depth travel experiences, unique personal insights, or current information about a destination, has a big impact on how potential tourists see and trust a destination (Rather et al., 2021; Ruan et al., 2024; Smets et al., 2021). This kind of content not only grabs and holds people’s attention, but it may also be as persuasive as traditional advertising (Wang and Huang, 2025).

The quality of user-generated content is widely recognized as important, yet most models dealing with it only define ways in which the direct effects can be verified or how these effects fit into traditional IQ (information quality) models and theories (Wang and Heckman, 1996). This ignores the fundamental change in the media ecology: now social media platform platforms are a “pseudo-environment” that fosters the construction of an urban image in the 21st century (Duffy et al., 2020). Some research suggests that users’ perception of destination as how appropriately its content aligned with their immediate task requirements and the users’ future task goals of the text-based-oriented information (Scholl-Grissemann et al., 2020; Rasoolimanesh et al., 2021). Later studies have found that several content dimensions affect the perceived destination image, such as value-added products (Zhang et al., 2025), degree of relevance to interests of users and search goals (Girija et al., 2023), recency of update, information completeness (Guan et al., 2023), and entertainment value (Girija et al., 2023). It is but to be expected when the use of social media is for practical as well as a pleasurable purpose and has been found to be the portal with the greatest linkage to the intention to visit (Gao et al., 2021; Chi and Phuong, 2022).

For Generation Z, content quality is no longer just a list of static information traits; it is also a moving force in the making of a mediated symbolic environment. High-quality content gives people information about the destination and changes how they see urban space by being useful, attractive, and relevant to the situation (Herfort et al., 2014). In this context, destination image is not solely formed through information dissemination; instead, it arises from a comprehensive process of media-environment construction.

This study utilises dual-process theories of information processing and insights from affective research to elucidate the mechanisms through which content quality may function within various psychological frameworks. Dual-process theory posits that the influence of content quality includes both systematic and heuristic processing (Chaiken, 1980). Information value, relevance, and completeness are expected to enhance the systematic nature of cognitive processing (Guan et al., 2023; Scholl-Grissemann et al., 2020). For instance, in short-video contexts, content that focuses on information may minimise perceived risk by delivering accurate and relevant references (Tellis et al., 2019). Conversely, presentation and aesthetic features can serve as heuristic cues that influence rapid decision-making (Zhang et al., 2025; Girija et al., 2023). Content that has a lot of emotion, through narrative structure, rhythm, and sensory stimulation, can make people feel things and have immersive experiences, which can get their attention and make them want to explore (Gao et al., 2021; Chi and Phuong, 2022). All of these patterns together show that high-quality material needs to match users’ expectations in both substance and presentation in order to help them create favourable cognitive and emotive images (Scholl-Grissemann et al., 2020).

Generation Z’s media intake is profoundly shaped by visual communication and multimedia interaction, rendering high-quality user-generated content essential in influencing both cognitive evaluations and emotional responses to metropolitan locales (Epafras et al., 2021; Liu et al., 2025). This kind of content can change how people think about, imagine, and feel about cities. These processes may ultimately be translated into visit intention (Wang X. et al., 2022; Wang Z. et al., 2022) and related forms of urban conative behaviour, including sharing and recommendation (Yoo and Kim, 2021). Yet existing studies have rarely examined the relative importance of the cognitive and affective pathways, or the ways in which they may operate together within a unified framework. Investigating how content quality influences urban image formation through real-time content perception (RTCP) and empathy may therefore provide a more nuanced understanding of this process and offer a stronger basis for the hypotheses developed in the present study.

### 2.3 Location-based user-generated content

Location-based user-generated content (LB-UGC) usually means content that people make themselves that includes information about where it is and makes geospatial data that people make (Zou et al., 2025; He et al., 2023). Locative media is now possible because of the rise of Web 2.0 technologies. These technologies have changed not only how people get information but also how people interact with urban space (Sui and Goodchild, 2011). This kind of content is a great way to learn about what users like, think, and have been through (Qiu et al., 2025; Fischer et al., 2012). LB-UGC gives people who are thinking about going on a journey particular reference points that help them plan their trip and choose a destination (Si et al., 2017). These reference points are based on written reviews, integrated rating systems, and advice from other travellers. An expanding body of empirical research demonstrates that participatory information systems profoundly influence tourist behaviour, frequently augmenting perceived trustworthiness and bolstering normative acceptance of featured destinations (Wang and Huang, 2025; Gretzel, 2011; Chen H. et al., 2025; Chen L. et al., 2025; Lu et al., 2014). In this context, LB-UGC serves as a unique analytical framework for researchers to delineate personal interest landscapes and examine the geographical aspects of consuming experiences (Liu et al., 2022; Fischer et al., 2012).

Despite the increasing scholarly focus, the literature on LB-UGC continues to exhibit a significant contradiction between cognitive and affective explanatory frameworks, revealing substantial deficiencies in existing theoretical narratives. A specific line of research, utilising information processing and decision-making models, asserts that the contextually situated information provided by LB-UGC alters behavioural intentions mainly through changes in cognitive appraisal (Scholl-Grissemann et al., 2020; Wang and Huang, 2025; Davras and Durgun, 2022; Lu and Huang, 2018). The explanatory burden is predominantly on evaluative judgement and rational inference. A parallel line of inquiry, rooted in sense-of-place theory and emotional geography, foregrounds the affective resonance embedded in geotagged narratives and place-linked imagery, arguing that destination-oriented behaviour is driven less by deliberative assessment than by emotional attachment and felt commitment to place (Xu et al., 2023; Espinoza-Figueroa et al., 2025; Kavouras, 2020). What remains insufficiently addressed, however, is the interplay between these two dimensions: most existing models either subordinate affect to cognition—treating emotional response as a downstream product of appraisal—or privilege a single psychological channel through which media influence is presumed to operate (Egger and Yu, 2022). Such uni-dimensional framing constrains our capacity to explain how LB-UGC shapes destination perception within digitally mediated, spatially embedded social environments (Rather et al., 2021; Lu et al., 2020).

To mitigate this limitation, the current study posits that LB-UGC may affect urban image via both cognitive appraisal and affective response. Its spatial embedding and contextual cues may assist users in assessing the relevance, utility, and accessibility of destination-related information, while place-linked narratives and visual content may enhance emotional engagement with the destination (Zou et al., 2025). We do not treat these processes as completely different here. Instead, they are seen as closely linked systems that help shape the image of a city through LB-UGC.

### 2.4 Electronic word-of-mouth

Electronic word-of-mouth (eWOM) is a crucial element in assessing locations and influencing travel decisions; it involves the voluntarily dissemination of consumer experiences (Ismagilova et al., 2021; Hennig-Thurau et al., 2004). Electronic word-of-mouth (eWOM), a prominent kind of online user-generated content (UGC), functions as a conduit for users to share their experiences and related perspectives with others (Tsao and Hsieh, 2015; Kwon and Ciechanover, 2017). The internet has significantly amplified word-of-mouth by enabling personal recommendations and experiences to disseminate to extensive audiences (Yang et al., 2024; Boo et al., 2022; Deng et al., 2024). In the tourism sector, electronic word-of-mouth (eWOM) has emerged as a pivotal source of information influencing individuals’ travel intentions and destination preferences (Rather et al., 2021; Ruan et al., 2024; Jiang et al., 2023; Zhu et al., 2023). This subjective information can be quite beneficial for those seeking to explore new locations, activities, and more nuanced travel-related subjects (Grewal et al., 2003; Söderlund and Rosengren, 2007). Recent studies on electronic word-of-mouth (eWOM) predominantly utilise a rational decision-making paradigm, viewing it primarily as a means of acquiring information. This perspective emphasises that electronic word-of-mouth (eWOM) influences cognitive assessment and decision-making by providing trustworthy and timely information that alleviates risk and uncertainty (Ye et al., 2023; Chen H. et al., 2025; Chen L. et al., 2025; Ruan et al., 2024; Gretzel and Yoo, 2008). It underscores users’ reliance on such information to alleviate perceived risk and uncertainty in decision-making processes (Chen H. et al., 2025; Chen L. et al., 2025; Wang and Huang, 2025). For example, keywords and viewpoints expressed by online opinion leaders, as types of electronic word-of-mouth (eWOM), can significantly enhance their impact on public perception (Ladhari and Michaud, 2015). This elucidates the increasing collaboration between marketers and content makers to engage customers on social media (Zeynep Bayazıt et al., 2017; Peralta, 2019; Lim and Rasul, 2022).

In contrast, another stream of research focuses on the affective and experiential dimensions of eWOM, proposing that it influences attitudes and behavioural intentions by eliciting emotional resonance and projection through narrative and entertaining content (Chen H. et al., 2025; Chen L. et al., 2025; Kim et al., 2024). For example, high-quality and high-credibility eWOM can evoke empathetic responses through contextual re-enactment and affective storytelling (Christou et al., 2025; Bai et al., 2025). eWOM content with entertainment value is also more effective in capturing user attention and fulfilling psychological needs such as hedonic gratification (Yang et al., 2024; Wang and Huang, 2025; Chen H. et al., 2025; Chen L. et al., 2025). Although both perspectives receive empirical support, existing studies often present them in parallel without examining clearly how they relate to one another. For example, eWOM can both enhance decision confidence and contribute to information overload. This suggests that its cognitive and affective effects are not fully separable in practice.

In this context, the current study examines eWOM as a stimulus that may affect urban image through cognitive appraisal and emotional response. On the other hand, narrative and emotionally laden eWOM may help people feel more connected to the place and understand it better. Examining these two pathways concurrently may enhance our comprehension of the impact of electronic word-of-mouth (eWOM) on urban perceptions.

### 2.5 The mediating role of real-time content perception

The location-based, ever-changing, and always-updated features of social media sites have made real-time information an important part of modern digital spaces (Kim, 2014). These capabilities enable users to share content instantaneously and to obtain real-time reviews, comments, and travel experiences as they arise (Roma and Aloini, 2019; Buhalis and Amaranggana, 2014; Yilmaz and Aytekin, 2018; Katyal and Sehgal, 2025). In this context, the present study characterises real-time content perception (RTCP) as a cognitive evaluation process by which consumers appraise the timeliness and contextual relevance of digital content (De Canio and Endrighi, 2024; Qiu et al., 2025). RTCP relates not only to the availability of real-time information but also to how users assess and analyse dynamic, spatiotemporally tagged content in social media environments (Kim, 2014; Roma and Aloini, 2019).

Existing research often treats real-timeness mainly as an external contextual attribute that triggers particular behavioural reactions. This does not sufficiently represent the intrinsic psychological mechanism by which individuals perpetually evaluate content immediacy and contextual relevance (Qiu et al., 2025; Liu et al., 2023). Consequently, RTCP is often positioned as a peripheral antecedent rather than recognised as an organism-level cognitive mechanism linking external stimuli to internal perception. This limits current understanding of the role RTCP may play in the formation of urban image.

Some studies have begun to explore how RTCP may influence consumption-related behaviour by identifying some of the psychological linkages involved. These studies suggest that the evaluation of time-sensitive content may reduce uncertainty, support situational judgment, and strengthen behavioural intention in digital consumption settings (Jiang et al., 2015; Li et al., 2021b; Fulkerson and Shank, 2000). In tourism contexts, such judgments may be especially important when users rely on social media to assess destinations, compare options, and form expectations.

Even so, RTCP remains insufficiently theorised as a distinct cognitive appraisal process within the SOR framework. In this study, it is therefore treated as a key mediating mechanism through which external content stimuli may influence urban image and subsequent urban conative behaviour.

### 2.6 The mediating role of empathy

This study defines empathy as a multifaceted psychological process wherein individuals emotionally connect with and cognitively comprehend the experiences, events, or symbolic meanings presented in new media content (Davis, 1983; Hosany et al., 2020). In the context of urban image perception, empathy includes feeling connected to people, places, and everyday life in the city, as well as putting oneself in someone else’s shoes and projecting oneself onto others (Li et al., 2025). Users may imagine the lifestyles and values associated with the city, as well as consider how they may live or engage with that urban environment. In this sense, empathy refers to the propensity to share and understand the emotional states of others while connecting them to one’s own interpretive.

Existing research on the mediating role of empathy in response to content stimuli has generally developed along two related lines. One emphasises absorption and emotional contagion. This study demonstrates that social media content, especially location-based user-generated content (LB-UGC), can amplify users’ sense of engagement through geotags, embodied narratives, and contextual storytelling, thereby creating an environment conducive to empathetic responses (Li et al., 2023; Xu and McGehee, 2012). Check-in photographs and detailed vlogs could enhance users’ sense of presence and emotional engagement with the location, hence stimulating empathetic processes (Xu et al., 2021; Guo et al., 2023). The alternate avenue of investigation underscores active interpretation. Rom this perspective, empathy arises not only from immersion but also from users’ imaginative involvement, perspective-taking, and cognitive engagement with the meanings conveyed by the information.

Consequently, this study recognises Empathy (E) as an essential mediating variable. Generation Z views empathy not as a passive emotional response but as an active affective process involving the interaction with environmental information and social cues (Dimock, 2019). Although it may involve cognitive engagement, its primary purpose in this study is to assess the emotional connection individuals establish in response to social media content. This mediating pathway helps us understand how social media changes the way Generation Z thinks and acts in cities.

### 2.7 Urban image

Urban has been a subject of research since the 1970s (Gunn et al., 1972; Hunt, 1971). It is a psychological construct that is usually thought of as the sum of all the beliefs, thoughts, and perceptions a person has about a location (Crompton, 1979). As theory has progressed, its multidimensional character has become widely recognised and generally includes the three basic aspects of cognitive, affective, and conative, which together form a comprehensive mental picture of a destination (Baloglu and McCleary, 1999; Tasci et al., 2007). This conventional understanding has provided the basis for measuring and researching the effect of destination image. Studies show that destination image shapes tourists’ perceptions and expectations (e.g., Baloglu and McCleary, 1999; Bigne et al., 2021; Guerreiro and Marques, 2020; Lalicic et al., 2021; Matos et al., 2015). It is established that internet-based content, particularly social media content, is considered one of the main sources of information that shapes the urban image of a tourist destination (Gartner, 1993; Matos et al., 2015; Tasci and Gartner, 2007; Lalicic et al., 2021). The activities of tourists on-site at the destination reshape pre-existing impressions and create an in-situ image (Abuhjeeleh et al., 2018; Chon, 1990; Kim, 2018; Matos et al., 2015; Vaughan and Edwards, 1999; Veloso et al., 2024). The emergence of digital media, especially the growth of social media platforms, is, however, radically reshaping the formation process and expression of urban image (Mak, 2017). Compared to the relatively fixed and individualised formation process in the traditional view, the online image of the city is becoming a dynamic, collaborative, and interactive construct (Lund, 2018; Wang and Chen, 2022). Even though the interconnection between tourism destination image and travel behaviour is well established (Echtner and Ritchie, 1993; Kim and Fesenmaier, 2017; Lalicic et al., 2021; Lao et al., 2023), there is still a lack of effective methods and tools to analyse the enormous amount of online information on social media platforms (Nayak et al., 2023). Although its importance remains widely recognised, the most appropriate methods of analysis and tools are still in the exploration and discussion phase (Bui and Tai, 2022; Jiang et al., 2021; Singgalen, 2024).

### 2.8 Urban conative behaviour

Conative response has long been recognised as an important component of destination image research, referring to an individual’s behavioural tendency or action readiness toward a place (Gartner, 1993). It is commonly reflected in pre-behavioural intentions such as willingness to visit, intention to recommend, positive word-of-mouth, and consumption-related commitment (Xue et al., 2022; Wang and Shahzad, 2024). In tourism studies, the conative dimension is often treated as the behavioural outcome of preceding cognitive and affective evaluations, linking destination perceptions to actual or intended actions.

Previous research suggests that cognitive image and affective image jointly shape conative outcomes (Zhang and Kim, 2023). In particular, affective attachment rooted in cultural identification, emotional connection, and sense of belonging plays a significant role in strengthening behavioural intentions toward destinations (Agapito et al., 2013). This indicates that conative behaviour is not formed independently, but emerges from the interaction between what individuals know or perceive about a place and how they emotionally relate to it.

In digital media environments, urban conative behaviour is increasingly influenced by social media-based destination experiences (Liang and Nguyen, 2024). Online content not only provides information for evaluating places, but also stimulates recommendation intentions, travel willingness, and other destination-related behavioural tendencies (Chen H. et al., 2025; Chen L. et al., 2025). Recent studies further suggest that users’ emotions and the urban image formed through social media significantly affect tourism behavioural intentions (Stylidis, 2018; Bao et al., 2019). For Generation Z in particular, whose destination-related decisions are often embedded in highly interactive and emotionally charged digital contexts, urban conative behaviour may be especially sensitive to both perceptual evaluation and empathic engagement.

Accordingly, this study conceptualises urban conative behaviour as the response outcome within the SOR framework. It captures the action-oriented tendencies that follow users’ cognitive and affective processing of social media content, and reflects how urban image and empathy are translated into destination-related behavioural intentions among Generation Z.

### 2.9 Research framework and hypotheses

Existing research has not fully explained how Generation Z forms urban image and develops destination-related behavioural tendencies through social media content. In particular, limited attention has been paid to the combined effects of multiple new media content attributes and the psychological mechanisms through which such effects unfold. Previous studies suggest that both content evaluation and psychological responses play an important role in shaping destination perception and behavioural intention (Rather et al., 2021; Baloglu and McCleary, 1999; Zhang and Wang, 2023). These findings underscore the necessity for a more cohesive framework. This study develops a model that synthesises content stimulation, psychological mediation, and behavioural response, grounded in the stimulation–Organism–Response (SOR) framework. Specifically, new media content quality, LB-UGC, and eWOM are treated as external stimuli; real-time content perception and empathy are positioned as key organism-level mechanisms; urban image is conceptualised as a perception-based outcome of this process; and urban conative behaviour is treated as the final response. The hypotheses are presented (see Table 1).

**Table 1:** Summary of research hypotheses.

Path type	Hypothesis	Statement
Stimulus→ mediator	H1a	Content quality has significant positive effect on real-time content perception.
	H1b	Content quality has significant positive effect on empathy.
	H2a	Location-based user-generated content has significant positive effect on real-time content perception.
	H2b	Location-based user-generated content has significant positive effect on empathy.
	H3a	Electronic word-of-mouth has significant positive effect on real-time content perception.
	H3b	Electronic word-of-mouth has significant positive effect on empathy.
Mediator → urban image/behaviour	H4a	Real-time content perception has significant positive effect on the conative dimension of Urban Image.
	H4b	Real-time content perception has significant positive effect on urban conative behaviour.
	H5a	Empathy has significant positive effect on the affective dimension of Urban Image.
	H5b	Empathy has significant positive effect on urban conative behaviour.
	H6	Urban Image has significant positive effect on urban conative behaviour.
Indirect effects on urban Image	H7a	Real-time content perception mediates the relationship between content quality and urban image
	H7b	Empathy mediates the relationship between content quality and urban image
	H9a	Real-time content perception mediates the relationship between location-based user-generated content and urban image
	H9b	Empathy mediates the relationship between location-based user-generated content and urban image
	H11a	Real-time content perception mediates the relationship between electronic word-of-mouth and Urban Image.
	H11b	Empathy mediates the relationship between electronic word-of-mouth and Urban Image.
Indirect and sequential effects on urban Conative behaviour	H8a	Real-time content perception mediates the relationship between content quality and urban conative behaviour.
	H8b	Empathy mediates the relationship between content quality and urban conative behaviour.
	H10a	Real-time content perception mediates the relationship between location-based user-generated content and urban conative behaviour.
	H10b	Empathy mediates the relationship between location-based user-generated content and urban conative behaviour.
	H12a	Real-time content perception mediates the relationship between electronic word-of-mouth and urban conative behaviour.
	H12b	Empathy mediates the relationship between electronic word-of-mouth and urban conative behaviour.
	H13a	Content quality has positive effect on urban conative behaviour through the sequential mediation of real-time content perception and Urban Image.
	H13b	Content quality has positive effect on urban conative behaviour through the sequential mediation of empathy and Urban Image.
	H14a	Location-based user-generated content has positive effect on urban conative behaviour through the sequential mediation of real-time content perception and Urban Image.
	H14b	Location-based user-generated content has positive effect on city-related conative behaviour through the sequential mediation of empathy and Urban Image.
	H15a	Electronic word-of-mouth has positive effect on urban conative behaviour through the sequential mediation of real-time content perception and Urban Image.
	H15b	Electronic word-of-mouth has positive effect on urban conative behaviour through the sequential mediation of empathy and Urban Image.

## 3 Methodology

### 3.1 Sample

**Figure 1:**
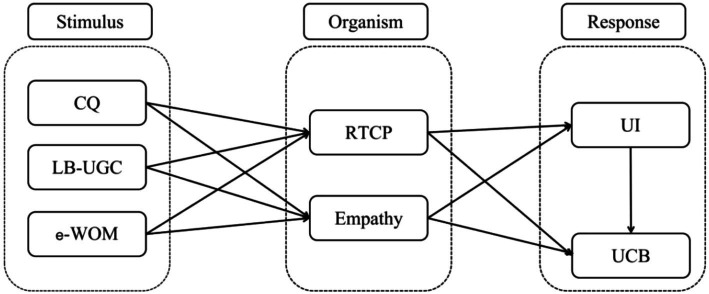
Theoretical model.

In this research purposive sampling was used to select the sample of the Generation Z social media users (born from 1997 to 2012), including users of Weibo and Xiaohongshu (Little Red Book) as some of the most popular platforms among this group of people in China. The questionnaires were sent to 20,000 qualified internet users (10,000 as Weibo users and 10,000 as Xiaohongshu users) who used the functionality of Weibo in the form of ‘@’ and Xiaohongshu in the form of direct messaging. To send invitations to participate in the survey, participants had to fulfil the following inclusion criteria: (1) born in 1997–2012, (2) showed great social media interest and regional identity. The initial group of valid respondents was 591, but having removed the cases of incomplete or invalid responses (the questionnaires having no data or multiple answers to the same question), the total sample consisted of 550 valid questionnaires (212 males, 338 females), and the effective response rate was 92.89%. In line with the methodological recommendations of Harlow and Ephross (1995) suggesting that SEM analysis necessitates 100–150 samples, our sample size (*N* = 550) is much higher; thus, it has strong statistical power to perform future analysis (see Figure 1).

To ensure that the empirical investigation matched the theoretical objectives of the study, purposive sampling was used to target Chinese Generation Z users who actively engage with urban-related content on social media platforms. The focus on Chinese Generation Z is analytical rather than merely practical. This group has a unique place in culture. For one thing, its members are digital natives who are quite familiar with worldwide trends in information, visual culture, and consumption. Conversely, they remain entrenched in a high-context and predominantly collectivist social milieu. This combination makes them especially useful for studying how social validation, symbolic cues, and communication with peers might help people feel empathy and change their plans to travel. These dynamics may be especially important in collectivist cultures, where group influence and social acceptability can have a big effect on how people make decisions.

The digital environment makes this concentration even more important (Campbell, 2020). Chinese Generation Z uses social media in a way that is very different from how it is used in many Western countries. Xiaohongshu, Douyin, and Weibo are not just places to get travel information. They mix social interaction, recommendation systems, visual storytelling, and ads in ways that change how people think about places before they go there. Consequently, Chinese Generation Z offers an optimal framework for examining the ways in which digital content characteristics elicit cognitive and emotional responses, and how these responses subsequently alter travel activity.

The strategy of this sampling technique was not to get a statistically representative sample of all of China’s Generation Z, but rather to get a theoretically relevant subpopulation that is deeply involved in how social media shapes urban images. We chose Weibo and Xiaohongshu as the major places to find participates because they are two of the most prominent and varied social media sites for Chinese Generation Z users. Weibo is basically a place for people to talk about things and share information with a lot of other people. Xiaohongshu, on the other hand, is all about sharing experiences, telling stories about lifestyles, and displaying cities based on where they are. When you use these platforms together, you have a better idea of how urban images are generated, understood, and shared in digital environments.

However, it is essential to acknowledge that targeted sampling through social media platforms may lead to particular forms of selection bias, specifically the under-representation of those with modest social media engagement or limited interest in urban tourist content. To assess the extent of potential bias, the demographic characteristics of the final sample were systematically compared with macro-level statistical data pertaining to Chinese Generation Z social media users obtained from credible sources. The observed similarities in age distribution, gender composition, and educational background offer empirical validation for a significant level of representativeness within the specified theoretical population. Nonetheless, despite these mitigation measures, non-users of the selected platforms and individuals who are largely disengaged from social media-based urban content may still be under-represented. Future studies may further enhance representativeness by employing stratified or quota sampling designs across multiple digital and non-digital channels (Zou et al., 2019).

To control sampling bias, especially the possibility of self-selection bias that is common in online surveys, such that there are systemic differences between the subjects and non-subjects, and this could lead to a biased response (Bethlehem, 2010), this study used several mitigation measures. The main strategy was simultaneous recruitment on two social media sites in an attempt to have a better diversified user base. Recruitment procedures (Xiaohongshu direct messaging and the use of the @ feature of Weibo) that are platform specific were utilised to target users with different levels of interest in the target topic, which minimises the tendency to recruit highly motivated users. Also, the Y = 30 compensation provision was made to attract the usual survey-averse user groups, which increased the heterogeneity of the sample.

In order to assess the representativeness of the sample and potential biases, the researchers compared the demographic characteristics of the final sample (e.g., age, gender, and educational background) against the macro-level statistical data on Chinese Generation Z social media users for the year 2023 (Tseng et al., 2024). The sample (38.55% male, 61.45% female; 53.64% undergraduate, 22.91% postgraduate) had a wide congruence with the population-wide trends, which gave empirical support to the representativeness. However, there are critical shortcomings that should be recognised: non-registered users of the target platforms and people who show total disinterest in tourism/urban imagery content might have been under-represented. Research done later should use stratified sampling or quota sampling designs to increase the representativeness of the population.

### 3.2 Measurement

In line with the extended Stimulus-Organism-Response framework, this study used structural equation modelling (SEM) as its main analytical method (Kock, 2017). The proposed model includes several forms of external content stimuli, parallel cognitive and affective mediating processes, and both direct and sequential effects on urban image and urban conative behaviour. SEM is well suited to this structure because it allows these interrelated relationships to be estimated simultaneously and makes it possible to compare the relative strength of different psychological pathways (Sarstedt et al., 2019).

The measurement items were primarily derived from known and validated scales (Zamora Lugo et al., 2025), but they were meticulously altered to align with both the theoretical framework of the study and the contextual environment of Chinese Generation Z. The goal was not to immediately apply conventional e-commerce or static website metrics to a novel context. Instead, the scales were improved to show how dynamic and socially mediated platforms like Xiaohongshu and Weibo are. For example, the items for location-based UGC and eWOM were adapted to capture platform-specific features such as geotags, immersive short-video storytelling, and real-time interactive comments, including bullet chats. Likewise, the items used to measure empathy were adjusted to reflect forms of cultural resonance and collective identification that are especially relevant in the Chinese context. This process of contextual adaptation strengthens the validity of the measurement design and helps ensure that the model operationalises the proposed cognitive-affective mechanisms in a way that suits a high-context digital environment.

Particular attention was given to the operationalisation of real-time content perception (RTCP) and empathy as organism-level constructs within the extended Stimulus-Organism-Response framework. RTCP was defined as an immediate cognitive appraisal of content timeliness, contextual relevance, and situational usefulness in information-rich digital settings. Empathy, by contrast, was defined as an affective resonance mechanism involving emotional projection, perspective-taking, and self-referential imagination prompted by narrative and immersive urban content (Zheng et al., 2022). These dimensions were included since they represent the main theoretical point of the study: Generation Z interacts with social media material through integrated cognitive and emotional processes.

The study also collected demographic information, including participants’ age, gender, and educational qualification. The latent variables in the model were content quality, LB-UGC, eWOM, real-time content perception, empathy, urban image, and urban conative behaviour, each measured through its associated observed indicators. All items were assessed using five-point Likert scales (Taherdoost, 2019). Table 2 provides brief definitions of these constructs and measurement items.

**Table 2:** Construct scale sources, and sample items.

Construct	Definition in this study	Measurement	No. of items	Source	Sample item
CQ	Users’ evaluation of the informational value, attractiveness, novelty, and completeness of destination-related content on social media platforms	Content quality was measured using five items assessing urban imagery on social media platforms, comprising two items for content innovation and three items for image appeal, with all items rated using five-point Likert scales 1 (strongly disagree) being counterbalanced by 5 (strongly agree).	5	Molinillo et al. (2020a,b)	Destination content obtained via Weibo/Xiaohongshu provides experiences that are dissimilar to traditional tourism promotions.
LB-UGC	Users’ perception of the usefulness and contextual value of geographically tagged user-generated content in providing location-specific information	LB-UGC was measured using three items to assess the attitudinal disposition of respondents towards spatially contextualised UGC, with all items rated using five-point Likert scales 1 (strongly disagree) being counterbalanced by 5 (strongly agree).	3	Khan A. et al. (2021), Khan M N. et al. (2021), and Khan M. et al. (2021)	I perceive user-generated content relevant to my geographical position as useful
EWoM	Users’ perception of the authenticity, trustworthiness, and influence of destination-related reviews and shared opinions on social media	Electronic word-of-mouth credibility was measured using three items assessing the extent to which users endorse the content posted on the platforms, with all items rated using five-point Likert scales 1 (strongly disagree) being counterbalanced by 5 (strongly agree).	3	Hennig-Thurau et al. (2004)	I perceive destination-related reviews and shares on Weibo/Xiaohongshu as authentic.
RTCP	Users’ immediate conative appraisal of the timeliness, situational relevance, and dynamic usefulness of destination-related content in social media environments	Three items conceptualised real-time perception, in which a five-point scale (1 = strongly disagree; 5 = strongly agree) was used to assess the instantaneous effectiveness of information processing.	3	Khan A. et al. (2021), Khan M N. et al. (2021), and Khan M. et al. (2021)	Content updates regarding target destinations on Weibo/Xiaohongshu occur with near synchronous immediacy.
Empathy	Users’ affective and perspective-taking response to urban content, including emotional resonance, cultural understanding, and self-projection into the destination context	The empathic responses were measured using four items (e.g., destination-focused short videos elicit visceral emotional fluctuations) where the probability scales were used, with 1 (very unlikely) to 5 (very likely).	4	Kozakevich Arbel et al. (2021)	I aspire to experientially immerse myself in this lifestyle through travel.
UI	Users’ conative and affective evaluation of the destination city as represented through social media content	Three affective image items and two conative image items were measured on five-point scales (1 = strongly disagree; 5 = strongly agree).	5	Hosany et al. (2020)	Contemplating visiting this city elicits anticipatory excitement.
UCB	Users’ behavioural intentions toward the destination, including visit intention, recommendation, and social sharing	Four items were measured, and the scale considered conative response: behavioural intention and advocacy behaviour, with the scales of 1 (never) to 5 (always) measuring behavioural enactment probability.	4	Chen et al. (2020)	Based on Weibo/Xiaohongshu content, I will include this city in my travel itinerary within 12 months.

A standard back-translation procedure was used to ensure that the questionnaire was clear and conceptually equivalent in Chinese (Brislin, 1986). First, a bilingual researcher translated the original scale into Chinese. Then, a second bilingual linguist translated the Chinese version back into English to make sure that the meaning of each item was still the same. This technique helped find wording errors and make sure that the two versions were more consistent with each other in terms of ideas.

Before the main survey, the questionnaire was pilot tested with 31 eligible Generation Z participants in Putian City, Fujian Province. The objective of the pilot study was to evaluate item clarity, identify potential interpretative challenges, and uncover deficiencies in the questionnaire design. Furthermore, three specialists with over a decade of expertise in media and cultural studies assessed the instrument to appraise its substance and phrasing. The definitive version of the questionnaire was amended based on expert feedback and pilot results.

To reduce the risk of common method bias, which can arise when data are collected through a single method such as self-report questionnaires, the study used both procedural and statistical remedies following Podsakoff et al. (2003). At the procedural level, respondent anonymity was protected to reduce social desirability bias. The questionnaire instructions were written clearly and stated that there were no right or wrong answers in order to encourage honest responses. Item order was randomised to minimise order effects and reduce patterned answering.

At the statistical level, Harman’s single-factor test showed that the first factor accounted for 32.4% of the total variance, which is below the commonly used 50% threshold. This suggests that common method bias was not a serious problem in the data. In addition, all constructs showed satisfactory internal consistency and convergent validity, with Cronbach’s alpha and composite reliability values above 0.80 and average variance extracted values above 0.50 (see Table 2). Taken together, these results suggest that common method bias was unlikely to have substantially distorted the findings.

## 4 Data results

### 4.1 Demographic results

Table 3 demonstrates the demographic characteristics of the participants. The participation of male participants in the sample is approximately 38.55%, and the remaining are female. The majority of the respondents possess a bachelor’s degree, which comprises 53.64, and 22.91% of the participants are postgraduate degree holders.

**Table 3:** Demographics of respondents (*N* = 550).

	*M*	Percent (%)
Gender		
Male	212	38.55
Female	338	61.45
Age		
18–19	178	32.36
20–22	237	43.09
23–25	135	24.55
Educational background		
High school and below	129	23.45
Undergraduate	295	53.64
Postgraduate	126	22.91

### 4.2 CFA test results

Confirmatory factor analysis (CFA) was used to examine the relationships between the observed variables and the latent constructs. Convergent validity refers to the extent to which the indicator variables measuring the same construct are highly correlated. Table 4 shows that the Cronbach’s alpha and composite reliability (CR) values for all constructs are above 0.80, while the factor loadings and average variance extracted (AVE) values are all above 0.50. This indicates that the constructs demonstrate satisfactory reliability and convergent validity (Taber, 2018).

**Table 4:** Factor loadings, CR, and AVE of items.

Constructs	Factor loading	CR	AVE
Content Quality (CQ)(Molinillo et al., 2020a,b)			
CQ1: The destination image content acquired via Weibo/Xiaohongshu is vibrant and novel	0.821	0.929	0.725
CQ2: Destination content obtained through Weibo/Xiaohongshu provides experiences divergent from conventional tourism promotions	0.875		
CQ3: Diverse representational formats of destination content on these platforms appeal to me	0.895		
CQ4: Destination content accessed on Weibo / Xiaohongshu demonstrates comprehensive coverage	0.837		
CQ5: Urban tourism content discovered on these platforms delivers unexpected delight	0.827		
Location-Based User-Generated Content (LB-UGC) (Khan A. et al., 2021; Khan M N. et al., 2021; Khan M. et al., 2021)			
LB-UGC1: Location-based UGC delivers valuable information, facilitating my acquisition of context-specific knowledge when needed	0.809	0.852	0.657
LB-UGC2: I perceive user-generated content relevant to my geographical position as useful (Adams and Janowicz, 2012)	0.804		
LB-UGC3: LB-UGC enables prompt and accurate provision of real-time location-specific supplementary information	0.82		
Electronic Word-of-Mouth (eWOM) (Hennig-Thurau et al., 2004)			
e-WOM1: I perceive destination-related reviews and shares on Weibo / Xiaohongshu as authentic	0.826	0.806	0.582
e-WOM2: I regard content creators disseminating urban information on these platforms as trustworthy	0.741		
e-WOM3: Information sourced from Weibo/Xiaohongshu exerts significant influence on my destination knowledge acquisition	0.717		
Real-Time Content Perception (RTCP) (Khan A. et al., 2021; Khan M N. et al., 2021; Khan M. et al., 2021)			
RTCP1: Content updates regarding target destinations on Weibo/Xiaohongshu occur with near-synchronous immediacy	0.88	0.847	0.65
RTCP2: Compared to traditional travel guides, new media platforms imbue destinations with vibrantly dynamic qualities	0.759		
RTCP3: Real-time content updates bridge psychological distance toward target destinations	0.775		
Empathy (E) (Kozakevich Arbel et al., 2021)			
E1: When viewing scenes of residents’ daily lives (e.g., markets, teahouses), I vicariously experience their joy	0.899	0.932	0.775
E2: Destination-focused short videos on social media trigger affective fluctuations	0.861		
E3: I comprehend the cultural signification embedded in local symbols (e.g., dialects, architectural styles)	0.871		
E4: I aspire to experientially immerse myself in this lifestyle through travel	0.89		
Urban Image (UI) (Hosany et al., 2020)			
UI1: The destination appears [dull 1-2-3-4-5 exciting] on social media	0.842	0.904	0.652
UI2: Contemplating visiting this city elicits anticipatory excitement	0.796		
UI3: It projects an ambiance of relaxed comfort	0.806		
UI4: Distinctive historical-cultural symbols characterize this urban representation	0.811		
UI5: Its visual identity demonstrates high distinctiveness in UGC	0.782		
Urban Conative Behaviour (UCB) (Chen et al., 2020)			
UCB1: Based on Weibo/Xiaohongshu content, I will include this city in my travel itinerary within 12 months	0.773	0.865	0.617
UCB2: I am willing to disseminate travel experiences about this city via social media	0.795		
UCB3: I would spontaneously add destinations to my trip due to real-time content exposure	0.805		
UCB4: I will recommend this city to friends and share customized itinerary templates	0.767		

SFL, standardized factor loadings; CR, composite reliability; AVE, average variance extracted.

The fit indices of the proposed measurement model were then examined in order to assess the adequacy of the model. Discriminant validity evaluates the extent to which different latent variables are empirically distinct from one another. In this study, discriminant validity was assessed using the average variance extracted (AVE) criterion (Steenkamp and Maydeu-Olivares, 2021). Discriminant validity is supported when the square root of the AVE for each construct is greater than its correlations with the other constructs (see Tables 4, 5).

**Table 5:** Correlations between variables.

	CQ	LB-UGC	eWOM	RTCP	E	UI	UCB
CQ	0.852						
LB-UGC	0.391***	0.811					
eWOM	0.441***	0.347***	0.763				
RTCP	0.493***	0.403***	0.419***	0.806			
E	0.482***	0.384***	0.563***	0.529***	0.881		
UI	0.425***	0.381***	0.382***	0.449***	0.460***	0.808	
UCB	0.520***	0.421***	0.482***	0.569***	0.555***	0.647***	0.785

### 4.3 Structural model testing

Model-fit statistics indicate an acceptable fit between the proposed structural model and the observed data (*χ*^2^/df = 1.644; RMSEA = 0.034; GFI = 0.936; CFI = 0.979; NFI = 0.949; TLI = 0.977; Table 6). These indices meet recommended thresholds and jointly suggest that the model provides a satisfactory representation of the data, supporting the adequacy of the model for hypothesis testing.

**Table 6:** Fit statistics for research model.

Fit index	Satisfied	Model
x^2^		509.655
RMSEA	0.00 ≤ RMSEA ≤ 0.08	0.034
GFI	0.85 ≤ GFI ≤ 1.00	0.936
CFI	0.85 ≤ CFI ≤ 1.00	0.979
NFI	0.85 ≤ NFI ≤ 1.00	0.949
TLI	0.85 ≤ TFI ≤ 1.00	0.977
x^2^/df	0.00 ≤ x2 /df ≤ 5.00	1.644

***p < 0.001.

### 4.4 Path coefficients and hypothesis testing

The structural model provides broad support for the hypothesised relationships among social media stimuli, the two organism-level variables, urban image, and urban conative behaviour. As shown in Table 7, all proposed paths are positive and statistically significant. In addition to statistical significance, the relative magnitude of the path coefficients shows meaningful differences across the cognitive and affective pathways.

**Table 7:** Standardized path coefficients to testing the causal effects of the constructs for model.

Hypothesis	Construct	Path	Construct	Estimate	S.E.	C.R.	p	Result
H1a	CQ	→	RTCP	0.326	0.056	6.569	***	Support
H1b	CQ	→	Empathy	0.248	0.052	5.492	***	Support
H2a	LB-UGC	→	RTCP	0.218	0.049	4.489	***	Support
H2b	LB-UGC	→	Empathy	0.157	0.046	3.535	***	Support
H3a	eWOM	→	RTCP	0.233	0.065	4.498	***	Support
H3b	eWOM	→	Empathy	0.415	0.065	8.179	***	Support
H4a	RTCP	→	UI	0.313	0.046	6.489	***	Support
H4b	RTCP	→	UCB	0.285	0.038	6.271	***	Support
H5a	Empathy	→	UI	0.318	0.043	6.884	***	Support
H5b	Empathy	→	UCB	0.238	0.035	5.56	***	Support
H6	UI	→	UCB	0.41	0.041	8.66	***	Support

***p < 0.001.

Among the stimulus variables, content quality (CQ) shows the strongest association with real-time content perception (RTCP) (*β* = 0.326, *p* < 0.001), whereas electronic word-of-mouth (eWOM) shows the strongest association with empathy (*β* = 0.415, *p* < 0.001). Location-based user-generated content (LB-UGC) is positively associated with both RTCP (*β* = 0.218, *p* < 0.001) and empathy (*β* = 0.157, *p* < 0.001). This results suggest that different kinds of social media stimuli are linked to different mental processes. Content that is rich in information and clearly presented seems to be more directly tied to quick cognitive appraisal. On the other hand, peer-generated evaluations and narrative content seem to be more closely related to emotive resonance.

Both organism-level variables have a favourable relationship with urban image (UI) and urban conative behaviour (UCB). Their effects on UI are almost identical in magnitude: RTCP has a path coefficient of β = 0.313, while empathy has a coefficient of *β* = 0.318, and both relationships are significant at *p* < 0.001. RTCP (*β* = 0.285, *p* < 0.001) and empathy (*β* = 0.238, *p* < 0.001) are also directly associated with UCB. In the final stage of the model, UI remains the strongest direct predictor of UCB (*β* = 0.410, *p* < 0.001).

Viewed as a whole, these results are better interpreted as evidence of differences in pathway strength rather than as unexpected associations. The findings do not challenge the broader destination image literature. Instead, they refine it by showing how different types of social media information are linked to different cognitive and affective pathways for Generation Z users.

### 4.5 Interpretation of direct effects and model robustness

Relative strength of the stimuli differs across the two organism pathways. For RTCP, content quality emerges as the dominant predictor (*β* = 0.326, *p* < 0.001), suggesting that when Gen Z perceives social-media travel content as accurate, complete, and well-presented, it most strongly enhances immediate cognitive appraisal. In contrast, LB-UGC (*β* = 0.218) and eWOM (*β* = 0.233) have comparatively smaller—though still meaningful—effects on RTCP.

For empathy, eWOM is the strongest predictor (*β* = 0.415, *p* < 0.001), with content quality (*β* = 0.248, *p* < 0.001) and LB-UGC (*β* = 0.157, *p* < 0.001) playing secondary roles. This indicates that peer assessments and socially integrated information significantly influence emotional engagement among Generation Z users.

Both organism-level variables contribute meaningfully to urban image formation. The near-identical effect sizes of RTCP (*β* = 0.313, *p* < 0.001) and empathy (*β* = 0.318, *p* < 0.001) suggest that cognitive and affective processes operate in parallel at this stage, jointly shaping how Generation Z constructs impressions of urban destinations. Urban image remains the strongest direct predictor of urban conative behaviour (*β* = 0.410, *p* < 0.001), although RTCP (*β* = 0.285, *p* < 0.001) and empathy (*β* = 0.238, *p* < 0.001) also retain direct effects.

**Figure 2:**
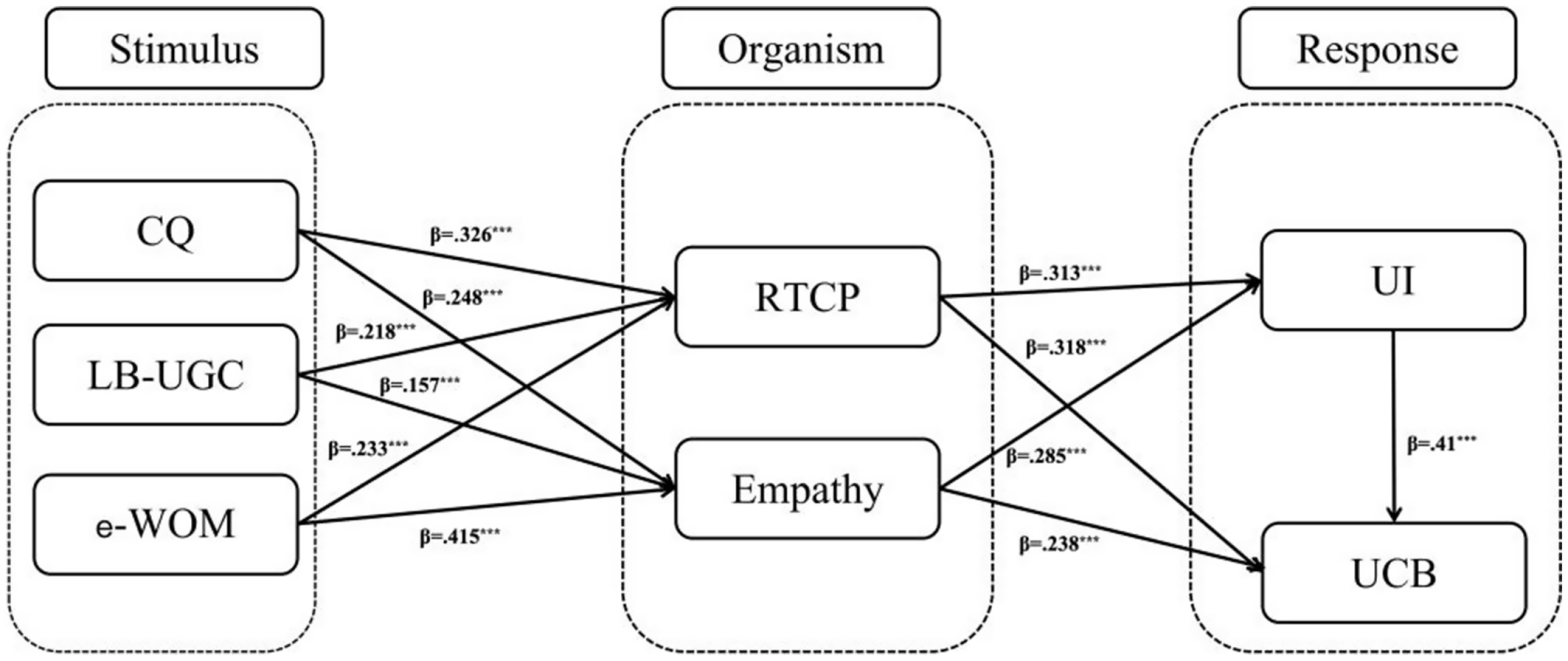
Results of structural equation model. ***p < 0.001.

**Figure 3:**
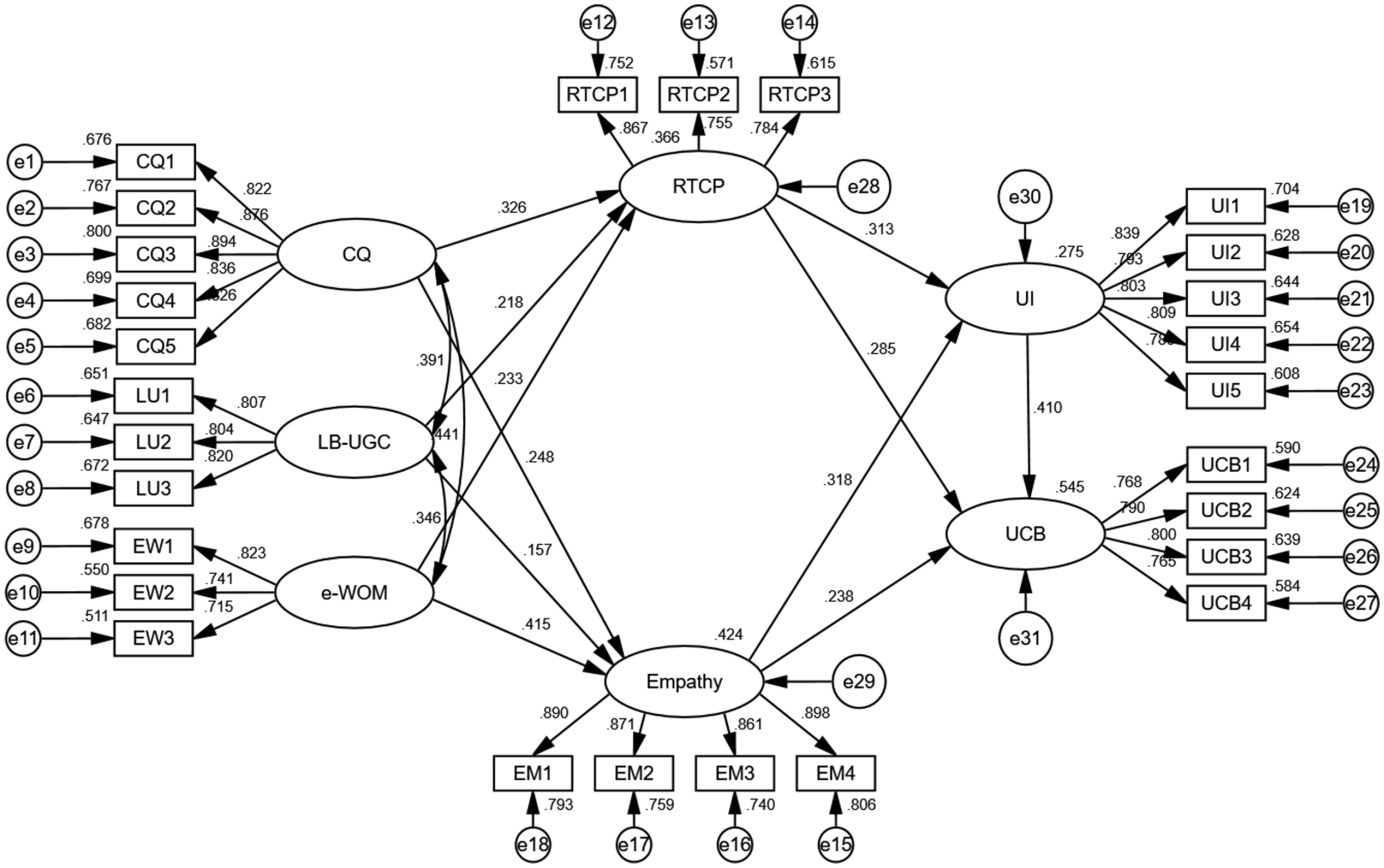
Fit statistics for model.

Taken together, these findings indicate differentiated pathway strength across the model. Content quality is more strongly associated with RTCP, whereas eWOM is more strongly associated with empathy (see Figures 2, 3).

### 4.6 Testing the mediating effects

To examine the indirect mechanisms in the proposed model, mediation effects were tested using bootstrapped bias-corrected 95% confidence intervals. As shown in Table 8, all hypothesised indirect effects are statistically significant, since none of the confidence intervals includes zero.

**Table 8:** Results of mediation analysis.

Hypothesis	Path	Effect	SE	Bias-corrected 95%CI		*p*	Result
				Lower	Upper		
H7a	CQ → RTCP → UII	0.102	0.029	0.055	0.174	0.001	Support
H7b	CQ → E → UI	0.079	0.025	0.037	0.137	0	Support
H8a	CQ → RTCP → UCB	0.093	0.026	0.052	0.156	0	Support
H8b	CQ → E → UCB	0.059	0.019	0.027	0.102	0.001	Support
H9a	LB-UGC → RTCP → UI	0.068	0.025	0.027	0.128	0.001	Support
H9b	LB-UGC → E → UI	0.05	0.02	0.018	0.099	0.003	Support
H10a	LB-UGC → RTCP → UCB	0.062	0.022	0.028	0.112	0	Support
H10b	LB-UGC → E → UCB	0.037	0.014	0.015	0.074	0.003	Support
H11a	eWOM → RTCP → UI	0.073	0.025	0.033	0.131	0.001	Support
H11b	eWOM → E → UI	0.132	0.031	0.081	0.204	0	Support
H12a	eWOM → RTCP→UCB	0.066	0.023	0.03	0.124	0.001	Support
H12b	eWOM → E → UCB	0.098	0.024	0.054	0.148	0.001	Support

For content quality (CQ), both mediating pathways through real-time content perception (RTCP) and empathy are significant for urban image (UI) and urban conative behaviour (UCB). The indirect effect of CQ on UI through RTCP is 0.102 [95% CI = (0.055, 0.174)], while the corresponding effect through empathy is 0.079 [95% CI = (0.037, 0.137)]. A similar pattern appears for UCB. The indirect effect of CQ on UCB through RTCP is 0.093 [95% CI = (0.052, 0.156)], compared with 0.059 [95% CI = (0.027, 0.102)] through empathy. Taken together, these findings suggest that the indirect influence of content quality is somewhat stronger through RTCP than through empathy.

For location-based user-generated content (LB-UGC), both mediators exert substantial indirect effects. The indirect effect of LB-UGC on UI through RTCP is 0.068 [95% CI = (0.027, 0.128)], while the same effect through empathy is 0.050 [95% CI = (0.018, 0.099)]. For UCB, the indirect effect through RTCP is 0.062 [95% CI = (0.028, 0.112)], while the effect through empathy is 0.037 [95% CI = (0.015, 0.074)]. This pattern shows that LB-UGC is connected to both cognitive and affective mediation. However, the RTCP route seems to be a little stronger in this group.

The pattern for electronic word-of-mouth (eWOM) differs. The indirect effect of eWOM on UI through RTCP is 0.073 [95% CI = (0.033, 0.131)], whereas the indirect effect through empathy is 0.132 [95% CI = (0.081, 0.204)]. The same tendency appears for UCB. The indirect effect of eWOM on UCB through RTCP is 0.066 [95% CI = (0.030, 0.124)], while the corresponding effect through empathy is 0.098 [95% CI = (0.054, 0.148)]. When compared to the other stimulus variables, eWOM has a stronger indirect link through empathy. This indicates that peer-generated assessments and narratives are more intimately associated with empathy than with RTCP alone.

In general, the mediation results demonstrate that both RTCP and empathy are important factors that connect social media stimuli to UI and UCB. The intensity of these indirect pathways fluctuates among different stimulus categories. CQ and LB-UGC show somewhat stronger indirect effects through RTCP, whereas eWOM shows stronger indirect effects through empathy. These differences suggest that different forms of social media content may activate different psychological routes in the formation of urban image and conative responses among Generation Z users.

### 4.7 Chained mediating effects

The serial mediation results provide further support for this pattern (see Table 9). For content quality and LB-UGC, the RTCP → UI pathway shows a stronger indirect effect on UCB than the empathy → UI pathway. This suggests that well-structured information and location-based cues are more likely to influence behavioural intention by first shaping conative appraisal and then strengthening urban image.

**Table 9:** Results of mediation analysis.

Hypothesis	Path	Effect	SE	Bias-corrected 95%CI		*p*	Result
				Lower	Upper		
H13a	CQ → RTCP → UI → UCB	0.042	0.012	0.022	0.071	0	Support
H13b	CQ → E → UI → UCB	0.032	0.011	0.015	0.061	0	Support
H14a	LB-UGC → RTCP → UI → UCB	0.028	0.011	0.012	0.055	0	Support
H14b	LB-UGC → E → UI → UCB	0.02	0.009	0.007	0.043	0.002	Support
H15a	eWOM → RTCP → UI → UCB	0.03	0.01	0.013	0.054	0.001	Support
H15b	eWOM → E → UI → UCB	0.054	0.015	0.031	0.09	0	Support

The serial mediation results provide further support for the differentiated psychological routes identified in the model (see Table 9). For content quality and LB-UGC, the indirect effect on urban conative behaviour is stronger through the RTCP → urban image pathway than through the empathy → urban image pathway. This suggests that structured information and location-based cues are more likely to influence behavioural response by first shaping users’ cognitive appraisal and then strengthening urban image.

For eWOM, the pattern is different. The indirect effect through the empathy → urban image pathway is stronger than that through the RTCP → urban image pathway. This indicates that peer evaluations and socially shared narratives exert their influence more strongly through affective engagement, which then contributes to urban image formation and subsequent urban conative behaviour.

In general, these serial mediation results suggest that different kinds of social media information affect the brain in diverse ways. It appears that informative and geographical signals primarily operate through RTCP, whereas socially evaluative content depends more on empathy. Urban image functions as a crucial downstream perceptual mechanism that links internal psychological processing to urban conative behaviour across several routes.

All of the proposed paths are statistically significant in this sample, but it does not mean the model is complete or can be used in every case. A model featuring numerous theoretically viable pathways may exhibit a good match with a singular dataset; yet, it necessitates additional validation across varied samples, platforms, and situations.

As a result, the interpretation gives more weight to the pattern of effects than to significance alone. The main conclusion is not just that all of the hypotheses are true. More importantly, it looks like different forms of social media stimuli work through different psychological pathways.

Content quality and LB-UGC have more impact when they go through RTCP. On the other hand, eWOM has a stronger effect when people can relate to it. This trend indicates that informational and location-based signals are more likely to alter urban image and urban conative behaviour via cognitive appraisal, while peer-generated assessments and social narratives have a more significant impact through affective involvement. The mediation results thus endorse a sequential process. Social media material affects RTCP and empathy. These internal responses subsequently influence urban image, which, in turn, reinforces urban conative behaviour. Urban image continues to be the most significant direct predictor of urban conative conduct, highlighting its pivotal role in the shift from internal psychological processing to behavioural response.

Simultaneously, the consistently prominent routes may suggest that the model has not yet fully encapsulated the complexity of the process. This overall trend aligns with previous studies about the impacts of content quality and electronic word-of-mouth in digital tourist contexts (Wong et al., 2022); yet, it may indicate that significant contingencies are still to be modelled. Travel involvement, cultural background, platform algorithms, and the intensity of social interaction may influence the strength of these relationships (Zhang et al., 2023). Subsequent study ought to evaluate the resilience of the suggested framework in various scenarios and with differing model specifications.

## 5 Discussion

### 5.1 The key findings

This study draws on the Stimulus-Organism-Response (SOR) framework to examine how features of new media content shape Generation Z’s urban image and subsequent behavioural responses. The findings suggest that content quality, location-based user-generated content, and electronic word-of-mouth function as key external stimuli. These stimuli influence urban image through two mediating mechanisms, real-time content perception and empathy, which in turn contribute to urban conative behaviour.

The results show that content quality strengthens real-time content perception, which then contributes positively to urban image and, ultimately, to urban conative behaviour. This finding is consistent with classic media effects research and information systems theory, both of which suggest that the objective qualities of information shape how users interpret content and make decisions (Wang and Heckman, 1996). High-quality information is more likely to encourage systematic processing and reduce uncertainty (Guan et al., 2023; Scholl-Grissemann et al., 2020). The present study confirms that content quality remains an important trigger of cognitive appraisal in social media settings.

At the same time, the results suggest that this cognitive route does not operate in isolation. IIts explanatory function contrasts with that of the affective route, diverging from previous research that regarded cognitive assessment as the primary predictor of behaviour (Quintal et al., 2010). It seems that the fast and changing nature of the digital information environment makes it less important for Generation Z to process information in a purely logical way. Cognitive assessment appears to serve primarily as an initial filtering procedure intricately associated with subsequent emotional responses. This aligns with the notion that Generation Z is adept at swiftly digesting fragmented information and utilising cognitive signals as an initial screening mechanism (Rodrigues et al., 2024). However, their choices seem to be heavily influenced by their immediate emotional state, since cognition and emotion work together to comprehend information (Zhang et al., 2025; Schreiner, 2022). It appears that urban conative behaviour arises not solely from cognition, but from the interplay between cognitive appraisal and affective reaction.

The findings also show that content quality can elicit empathy, which in turn shapes urban image and influences behavioural intention. This result supports affective processing theory and narrative communication research, both of which suggest that the emotional value and narrative form of content can trigger empathic engagement among users (Hosany et al., 2020). The significant mediating role of empathy provides direct empirical support for the view that affective response is not simply a secondary outcome of cognitive evaluation. Rather, it appears to operate as an important mechanism in its own right, with the capacity to contribute directly to behavioural conversion in visual social media environments.

### 5.2 Theoretical implications

#### 5.2.1 Validation of stimulus mechanisms

The theoretical implications of this study are best understood through the combined operation of the two pathways. Content quality (CQ) appears to activate cognitive and affective processing simultaneously, with both routes contributing to urban image (UI) and urban conative behaviour (UCB). This finding moves beyond the familiar debate in earlier literature over whether behaviour is driven mainly by cognition or mainly by affect (Cheng, 2024). Traditional destination image theory, for example, distinguishes between cognitive and affective image and argues that both contribute to overall destination image, but it often presents their relationship in broadly sequential terms (Baloglu and McCleary, 1999). By contrast, the present model suggests that, for Generation Z, real-time content perception (RTCP) and empathy triggered by CQ emerge almost simultaneously and interact dynamically before contributing to urban conative behaviour.

The process is therefore not adequately described as a simple cognition-affection-conation sequence. Instead, it reflects a more immediate and overlapping psychological response. In this respect, the study offers a useful refinement of both the classic SOR model and destination image theory in digitally mediated environments. It suggests that, for Generation Z, high-quality content must function at once as a source of information and as an affective medium if it is to shape urban image and influence urban conative behaviour effectively.

The theoretical importance of location-based user-generated content (LB-UGC) lies in showing how geo-mediated content can function at the same time as a cognitive resource and an affective vehicle. The empirical results support all hypotheses linking LB-UGC to UI and UCB through both RTCP and empathy. This matters because prior research has often treated these functions separately. One line of work has emphasised the cognitive and instrumental value of LB-UGC, arguing that geotags and contextualised information reduce uncertainty and improve travel planning (Scholl-Grissemann et al., 2020; Davras and Durgun, 2022). The present findings are consistent with that view. LB-UGC significantly enhances RTCP, suggesting that precise location cues and immediate scene information act much like a dynamic cognitive map. They increase credibility and strengthen users’ sense of presence, thereby supporting the formation of a more concrete and reliable urban image (Gao and Li, 2022; Yin et al., 2023). In this sense, LB-UGC performs a spatial anchoring function that reinforces cognitive understanding of place.

At the same time, the study also supports research that highlights the affective power of LB-UGC in generating place attachment and emotional identification (Xu et al., 2023; Kavouras, 2020). The findings indicate that LB-UGC can successfully evoke empathy. Users seem to feel a kind of vicarious presence when they see tailored stories linked to certain areas. This diminishes psychological distance and enhances emotional projection, rendering it more immediate and vivid (Kim et al., 2020). This technique makes location-based storytelling more than just a way to tell people about a place. It also encourages people to picture themselves in it, which makes them feel more connected to the city.

The primary theoretical contribution is not in the independent validation of these two pathways, but in demonstrating their collaborative functioning. The integrated model posits that LB-UGC can affect urban conative behaviour via RTCP and simultaneously influence urban conative behaviour directly or indirectly through empathy. So, for people in Generation Z, a brief video or post with a geotag may serve two purposes at once. It might be valuable as a reference because it has relevant and reliable information, and it might also make people feel something right away because of the tale it tells. This intersection of cognition and affect questions the reductive premises of prior research, which frequently prioritised either instrumental rationality or affective experience, but not their conjunction.

The serial mediation results elucidate this mechanism further. Whether LB-UGC functions via cognitive evaluation or affective reaction, its impact seems to be solidified through urban imagery before it manifests as consistent urban conative activity. This suggests that even highly contextualised and instantaneous digital input necessitates internal interpretation and meaning construction to exert a more profound influence on conduct. Consequently, LB-UGC is more accurately characterised not merely as an informative cue or an emotional trigger, but as a composite stimulus that offers both the spatial framework essential for decision-making and the immersive narrative context that fosters emotional engagement. In digital environments, its role in urban image formation is therefore more multidimensional and dynamic than earlier theories have often suggested.

Electronic word-of-mouth (eWOM), as a distinctly social form of stimulus, provides a particularly strong illustration of the interaction between cognitive trust and affective response. The results support all hypotheses linking eWOM to UI and UCB through both RTCP and empathy. These findings matter because research on eWOM has also been divided in emphasis. One body of work has approached eWOM from a rational decision-making perspective, treating it as a credible source of experiential information that reduces risk and uncertainty and thereby improves cognitive evaluation (Ye et al., 2023). The present findings broadly support this interpretation. Credible eWOM strengthens users’ trust in real-time information and increases RTCP, suggesting that peer-generated content improves the perceived value of dynamic destination information by giving it social validation (Wu et al., 2024).

A second body of research has focused more on the affective and experiential dimensions of eWOM, arguing that emotionally rich narratives and personal accounts influence behaviour by eliciting empathy and emotional projection (Chen H. et al., 2025; Chen L. et al., 2025). The current study provides especially strong support for this view. The affective pathway is particularly prominent in the case of eWOM. Emotional expressions and shared sentiments embedded in peer communication appear to trigger empathy directly (Qiu et al., 2023). When users read others’ enthusiastic or emotionally charged travel accounts, they are not only receiving information but also entering into an emotional relation with the experience being described. This empathic engagement can translate into favourable feelings towards the destination and, in some cases, into an immediate desire to visit or share.

The findings have larger theoretical implications since they illustrate that both routes function within a singular paradigm, while also indicating that the empathy pathway is particularly robust for electronic word-of-mouth (eWOM). This indicates that, for Generation Z, the persuasive efficacy of peer-generated communication may rely not solely on the conveyed information, but also, potentially more significantly, on the social and emotional energy inherent within it. In this regard, eWOM may influence urban conative behaviour not merely by disseminating information, but by fostering a sense of connection to a socially endorsed emotional response.

This may be especially important in China. The considerable influence of eWOM on empathy (*β* = 0.415) can be comprehended through a cultural perspective. In a collectivist society such as China, digital behaviour is often shaped by peer approval, social conformity, and collective symbolic importance (Li et al., 2024). Generation Z in China may be more vulnerable to emotional contagion and social proof in online groups compared to individuals in more individualistic settings. On sites like Xiaohongshu, eWOM is not only a source of information, but it also acts as a way to get social approval that makes emotional responses stronger. High-context communication, characterised by recognisable symbols that evoke shared memories, identities, and feelings of belonging, may enhance the emotional impact of urban imagery.

The results of the serial mediation fill in the gaps by showing how social stimuli are taken in and turned into behaviour. eWOM seems to start by making people think and feel things on their own. These responses then help to create a bigger picture of the city, which then affects how people act in the city. This indicates that tourism choices within social media contexts are not merely individual preferences developed in isolation. They are also shaped by social norms, collective meanings, and culturally embedded forms of emotional resonance. The theoretical contribution of this study, therefore, is not only to confirm that cognition and affect both matter, but to show how they interact differently across stimulus types and how this interaction helps explain urban image formation and urban conative behaviour among Generation Z in a digital and culturally specific environment.

#### 5.2.2 Experiential behaviour drivers

The main theoretical contribution of this study is the development of a dual-mediation model of urban image formation among digital natives, which extends the traditional Stimulus-Organism-Response framework in an important way. Although the SOR model has been widely used in tourism and media research, previous studies have tended to examine either cognitive appraisal (De Canio and Endrighi, 2024) or affective experience (Hosany et al., 2020) separately. Much less attention has been given to how these two processes operate together within the same model, or to their relative explanatory strength.

This study demonstrates that cognitive and affective processes function concurrently within a dual-pathway framework, rather than as discrete entities. In the realm of urban tourist choices among Generation Z, the affective pathway, facilitated by empathy, seems to possess greater explanatory efficacy than the cognitive pathway in isolation. This conclusion corroborates recent assertions that affect is crucial in digital decision-making (Rodrigues et al., 2024). It also contributes to the existing body of research by concentrating on empathy as a more specific psychological process rather than merely a positive emotion. Empathy is particularly pertinent in this context as it encompasses psychological simulation, perspective-taking, and vicarious experience. In that regard, it provides a more accurate depiction of user interaction with social media content than general concepts of affect alone.

The results also build on past studies that mostly looked at the quality of static information (Ismagilova et al., 2020). They propose that in dynamic digital contexts, the temporal aspect of content is significant in addition to its spatial aspect (Yang et al., 2025). Users seem to judge information not just by what it shows and where it is, but also by how up-to-date and relevant it looks to be given the context. This elucidates the significance of real-time content perception as a crucial component of the cognitive pathway in the model.

The results also build on past studies that mostly looked at the quality of static information (Ismagilova et al., 2020). They propose that in dynamic digital contexts, the temporal aspect of content is significant in addition to its spatial aspect. Users seem to judge information not just by what it shows and where it is, but also by how up-to-date and relevant it looks to be given the context. This elucidates the significance of real-time content perception as a crucial component of the cognitive pathway in the model.

In a broader context, the results suggest that the interaction between cognitive evaluation and emotional response may facilitate the shift from image creation to urban conative behaviour. This is consistent with affective bond theory, which argues that emotional attachment can strengthen behavioural commitment (Agapito et al., 2013). In particular, the mediating role of empathy highlights the transformative power of narrative content. Building on work on media narratives and destination affective image (Chen H. et al., 2025; Chen L. et al., 2025), this study shows that empathy is a key psychological mechanism linking contextualised narratives to affective identification and, in turn, to urban conative behaviour. This offers a more detailed explanation of the emotionally driven decision patterns often associated with Generation Z in digital environments.

#### 5.2.3 Dual mediation approaches

This integrated analytical framework elucidates the distinct functions of the two processes. Empathy serves both as a mediating variable and as a mechanism that seemingly expedites the transition from conative assessment to affective attachment in environments influenced by real-time and socially mediated material. Simultaneously, RTCP and empathy function concurrently, indicating a dual-processing framework that is especially pertinent to digital-native consumers. This offers a more sophisticated process-oriented elucidation of how urban imagery is constructed within modern social media contexts.

#### 5.2.4 Cultural specificity and generalisability

This study examines Chinese Generation Z as a significant cohort whose psychological interpretation of urban images is influenced by extensive social media usage, short-video consumption, and culturally ingrained emotional engagement methods. In the Chinese sociocultural setting, collectivist tendencies, narrative identification, and socially mediated emotional resonance may enhance the influence of empathy on the formation of affective urban images and subsequent behavioural intentions. The contextual qualities offer a significant interpretative framework for comprehending the considerable explanatory power of empathy evident in the empirical findings.

The limitations of generalisability must be acknowledged. The structural logic of dual conative-affective mediation may be applicable to other digital-native populations; however, the prominence of empathy-based pathways is expected to differ among civilisations with varying cultural norms, media ecologies, and platform affordances. Consequently, these findings must be evaluated within defined parameters, and subsequent research should evaluate the suggested model across varied cultural contexts to determine its wider relevance.

### 5.3 Practical implications

The findings of this study have direct practical implications for city branding and social media content design. The results suggest that content features shape urban image and conative behaviour through two main mechanisms: real-time content perception and empathy. In practical terms, this means that destination strategies should focus not only on what content says, but also on how it is presented, how frequently it is updated, and how it circulates socially on the platforms used by Generation Z.

First, platform managers and tourism marketers should make greater use of location-based and real-time content (Citaristi, 2022). Destination posts will probably work better if they are linked to specific places, things that are going on right now, and time-sensitive information, rather than just static promotional descriptions. For example, a city tourism account might post short videos of well-known places and update them during local events, night markets, festivals, or seasonal activities (Wu and Lai, 2024). These posts could have geotags, simple directions for getting there, and real-time participation cues like where to go, what’s going on right now, and what visitors can do while they are there. This kind of design is more likely to make content more relevant to the context and improve how people perceive it in real time. The results show that this is an important factor in how urban images are formed and how people react.

Second, destination content should focus more on stories that make people feel something. If you only use facts to communicate with Generation Z, it’s not likely to work. It works better when it includes everyday urban settings, personal experiences, and stories that are based in the area. One useful example would be a short video campaign based on stories from people who live in or visit certain neighbourhoods, cafés, bookshops, markets, or streets. This type of content is more likely to make people feel empathy because it shows the city through real-life experiences instead of just abstract destination labels. The findings indicate that empathy is a significant mechanism by which social media content influences the emotional aspects of urban imagery and fosters subsequent behavioural intentions.

Third, tourism marketers should come up with structured rewards for user-generated content that combines geotagging with participation in a theme. Instead of just asking users to post travel photos, platforms could set up themed content tasks like hidden local food routes, one-day neighbourhood walks, or city memory spots and ask users to upload short posts with location tags and their own thoughts (Makhal et al., 2023). Then, official city or tourism accounts could repost some of the best posts. This method would make real place-based content more visible and increase the number of urban stories that are socially credible. It would also help link official communication with grassroots expression. This is especially important for getting Generation Z users involved, since they often rely on content made by their peers when making travel plans.

All of these implications point to three practical priorities for organising urban digital communication: timely updates linked to locations, emotionally engaging stories, and guided user participation. These are real ways for cities to improve their branding efforts with Generation Z on social media.

### 5.4 Limitations and future research

This study should be understood within several clear boundaries. First, it focuses on Generation Z in the Chinese sociocultural context, and the findings need to be interpreted in light of that setting. Chinese Generation Z is a theoretically meaningful cohort because its members are shaped by intensive social media use, short-video consumption, and culturally embedded forms of emotional engagement. Within this context, collectivist orientations, narrative-based identification, and socially mediated emotional resonance may strengthen the role of empathy in shaping urban image and subsequent behavioural intention. These contextual conditions provide an important lens for interpreting the relatively strong explanatory power of empathy in the present findings.

At the same time, this cultural and technological specificity limits broader generalisation. Although the overall logic of dual cognitive-affective mediation may remain relevant to other digital-native populations, the relative strength of empathy-based pathways is likely to vary across societies with different cultural norms, media environments, and platform structures. In particular, the strong affective pathway found here may be partly amplified by the collectivist character of Chinese society. The findings may therefore not transfer directly to more individualistic settings, where cognitive appraisal could play a more prominent role in destination choice. A similar caution applies across age groups. Generation Z’s high level of digital immersion and tendency towards rapid, media-driven decision-making distinguish it from older cohorts, which limits the extent to which the results can be generalised beyond this generation.

Second, the sample consists mainly of Generation Z users who are active on major social media platforms. The results may therefore be less applicable to individuals with lower levels of digital engagement or to those who do not use such platforms at all. The research design attempted to reduce this problem by drawing participants from two different platforms, Weibo and Xiaohongshu, which have different user profiles, and by using randomisation strategies and appropriate incentives during questionnaire distribution to encourage participation from users with varying levels of willingness to respond. These steps were intended to improve sample diversity and representativeness. Even so, the sample still mainly reflects users with relatively strong digital skills and high social media activity. As a result, digitally marginalised groups are likely to be underrepresented.

Third, the measurement design is based mainly on current mainstream content formats and established psychological scales. It may therefore not fully capture the influence of newer media forms, including artificially generated content. In addition, because the study relies on self-report survey data analysed through structural equation modelling, common method bias remains a possible concern. Procedural remedies, including respondent anonymity, were used during data collection to reduce this risk, but reliance on self-reported measures remains a methodological limitation (Hu, 2018). Fourth, the use of cross-sectional data means that the study cannot trace how urban image perception develops or changes over time.

These limitations suggest several directions for future research. First, cross-cultural comparative studies and multi-group analyses across generations, such as Millennials and Generation X, would help test the generalisability of the model and examine whether the role of empathy differs across cultural settings, media systems, and age cohorts. Second, longitudinal designs would make it possible to track changes in urban image perception and behavioural intention over time, particularly before and after major events or destination marketing campaigns. Third, future research could strengthen the methodological design by combining survey data with physiological measures such as eye tracking, which may offer a more objective view of attentional patterns and the processing of visual urban information (Daikeler et al., 2020). Finally, as metaverse technologies continue to develop and virtual-physical environments become more integrated, research on image perception and tourism behaviour in metaverse settings is likely to become an especially valuable area for further study (Dang Quan et al., 2024).

## 6 Conclusion

This research examines the mechanisms by which characteristics of new media content affect Urban Image (UI) perception and behavioural intentions within Generation Z. The results indicate that Content Quality (CQ), Location-Based User-Generated Content (LB-UGC), and Electronic Word-of-Mouth (eWOM) substantially influence urban image and urban conative behaviour via the dual mediation of Real-Time Content Perception (RTCP) and Empathy (E). The results indicate the significance of both cognitive appraisal and affective response in influencing urban image and urban conative behaviour. In this dual-pathway structure, empathy is a key part of the affective pathway, and RTCP is a key part of the cognitive pathway. But their relative influence changes depending on the type of stimulus. Content quality and LB-UGC have a stronger connection to RTCP, while eWOM has a stronger connection to empathy. These results add a more context-sensitive angle to studies on how cities communicate and market tourism in the digital age. They enhance our comprehension of the psychological mechanisms that drive media effects and provide theoretically informed and practical insights for city branding strategies designed to engage younger demographics.

A notable theoretical advancement of this study is the contextualised refinement of the traditional SOR model, accompanied by a more comprehensive explanation of its operations within a social media framework. It not only interacts with the theoretical discussion regarding the dominance of conative versus affective pathways in digital communication—showing that for Generation Z, the affective empathy pathway is more effective at explaining things—but it also, and more importantly, moves theory forward by creating and supporting a dual mediation model. This concept integrates real-time conative evaluation, emphasising the timing and context of information, with empathic reactions that concentrate on emotional projection and narrative immersion. This theoretical advancement establishes a more defined psychological framework for understanding the influence of social media on place identity and introduces an analytical model to shift related research from investigating “whether an influence exists” to examining “how the influence manifests.”

This research employed a rigorous empirical methodology to evaluate the theoretical predictions; yet, several limitations persist. The research sample primarily focused on active social media users in China, requiring more examination to evaluate the cross-cultural applicability of the results. Cross-sectional data can show how variables are related, but it cannot show how image perception and behavioural intentions change over time. Moreover, the measurement tools were predominantly derived from contemporary popular content formats; their relevance to nascent formats, such as automatically generated material, necessitates further investigation.

Based on the contributions and limits indicated above, future study could go in a number of different areas. First, doing cross-cultural comparative research would assist see how this model works in different sociocultural settings and what factors affect it. Secondly, longitudinal tracking or experimental methodologies would enable researchers to dynamically analyse the evolving trajectories of users’ psychological mediating processes and behavioural intents preceding and following significant events or marketing efforts. Furthermore, the progression of technologies like the metaverse and mixed reality will facilitate the exploration of methods for constructing a sense of place and enhancing human-computer interaction inside integrated virtual-physical environments, emerging as a significant frontier (de Oliveira and Painho, 2021).

## Data Availability

The raw data supporting the conclusions of this article will be made available by the authors, without undue reservation.
